# Supplementary material for: MetaQTL: a package of new computational methods for the meta-analysis of QTL mapping experiments
Source: BMC Bioinformatics. 2007 Feb 8;8:49. doi: 10.1186/1471-2105-8-49 (PMC1808479; doi:10.1186/1471-2105-8-49)
Supplement: Additional File 7 — MetaQTL Package : jar file and tutorial. This Zip archive contains both the MetaQTL JAR file and the files of the tutorial. [file 1471-2105-8-49-S7.zip › org.inra.metaqtl/doc/org/metaqtl/class-use/QtlPartition.html]

Uses of Class org.metaqtl.QtlPartition


|  |  |  |
| --- | --- | --- |
| **Overview** \| **Package** \| **Class** \| **Use** \| **Tree** \| **Deprecated** \| **Index** \| **Help** | |  |
| PREV   NEXT | **FRAMES**    **NO FRAMES**     **All Classes** |  |


---


## **Uses of Class org.metaqtl.QtlPartition**

| Packages that use QtlPartition | |
| --- | --- |
| **org.metaqtl.adapter** |  |
| **org.metaqtl.graph** |  |
| **org.metaqtl.main** |  |

Uses of QtlPartition in org.metaqtl.adapter

| Methods in org.metaqtl.adapter that return QtlPartition | |
| --- | --- |
| `static QtlPartition` | `QtlPartitionAdapter.adapt(MetaQtlAnalysis analysis, java.lang.String chrom, java.lang.String trait, int k)`             Adapt the `MetaQtlAnalysis` `analysis` for a given value of the number of clusters `k` and a given trait name `code`. |
| `static QtlPartition` | `QtlPartitionAdapter.adapt(MetaQtlData qtlData)`             Adapt the given `MetaQtlData` into a `QtlPartition` and returns it. |
| `static QtlPartition` | `QtlPartitionAdapter.adapt(MetaQtlResult result, Qtl[] qtls, int k)` |

Uses of QtlPartition in org.metaqtl.graph

| Fields in org.metaqtl.graph declared as QtlPartition | |
| --- | --- |
| `QtlPartition` | `ChromGraph.qtlPart`             The Qtl partition |

Uses of QtlPartition in org.metaqtl.main

| Methods in org.metaqtl.main that return QtlPartition | |
| --- | --- |
| `static QtlPartition` | `MetaMain.getQtlPartition(Chromosome chromosome, IBioOntology ontology)` |
| `static QtlPartition` | `MetaMain.getQtlPartition(Chromosome chrom, MetaQtlAnalysis analysis, java.lang.String trait, int k, char tclust)` |

---


|  |  |  |
| --- | --- | --- |
| **Overview** \| **Package** \| **Class** \| **Use** \| **Tree** \| **Deprecated** \| **Index** \| **Help** | |  |
| PREV   NEXT | **FRAMES**    **NO FRAMES**     **All Classes** |  |


---
